# Male genital self-image, premature ejaculation, and affecting factors

**DOI:** 10.1093/sexmed/qfae041

**Published:** 2024-07-08

**Authors:** Vesile Koçak, Osman Tufan

**Affiliations:** Obstetrics and Gynecology Nursing, Nursing Faculty, Necmettin Erbakan University, Konya, Türkiye; Urology, Dr Vefa Tanır Ilgın State Hospital, Konya, Türkiye

**Keywords:** Keywords: male, premature ejaculation, genital self-image

## Abstract

**Background:**

Negative genital self-image is associated with sexual unresponsiveness and dysfunction.

**Aim:**

This study aims to determine the relationship between men’s genital self-image and premature ejaculation, with identifying influencing factors.

**Methods:**

The research is designed as a descriptive and correlational study. The sample consists of 188 men aged 18 to 60 years who volunteered to participate in the study.

**Outcomes:**

A negative correlation was observed between genital self-image and premature ejaculation (*P* < .05).

**Results:**

The average age of the participating men was 39.5 ± 9.79 years (mean ± SD), with 91.5% being married. The average age of the participants’ first sexual encounter was 20.43 ± 4.01 years, and 38.3% reported experiencing premature ejaculation. The mean score for the Male Genital Self-image Scale was 21.10 ± 5.59, and that for the Premature Ejaculation Diagnostic Tool was 6.96 ± 3.73. As a result of the study, it was revealed that participants who experienced premature ejaculation were not at peace with their bodies and were dissatisfied with their sexual experiences and their genital size and function, with significantly lower levels of genital self-image (*P* < .05).

**Clinical Implications:**

Identifying factors that affect men’s genital self-image is crucial for maintaining sexual functions.

**Strengths and Limitations:**

In Turkey, sexuality is a taboo subject, often considered shameful and rarely discussed, making it challenging to find participants willing to engage in research.

**Conclusion:**

Men’s genital self-image influences the characteristics of premature ejaculation.

## Introduction

Genital self-image, a crucial aspect of body image, refers to individuals’ emotional-psychological perception and awareness of their sexual organs in terms of sexual function and satisfaction.^1^ Research has demonstrated that subjective experiences and the enjoyment of sexuality are affected by genital self-image. A negative genital self-image may result in sexual unresponsiveness and dysfunction, encompassing issues such as sexual avoidance, shame, anxiety, and dissatisfaction with sexual activity, and male genital satisfaction is an important aspect of psychosocial and sexual health.^2^^,^^3^

Since ancient times, the size of the penis has been a significant concern for men.^4^ Similarly, in contemporary culture, the meaning attributed to the penis plays a fundamental role in defining male identity. Consequently, penis size and shape remain crucial concerns for many men.^5^ Among men, discussions about penis measurement are widespread. Perceiving one’s sexual organs to be below ideal standards can jeopardize a man’s self-confidence. Some men experience distress whether they have a micropenis or even an average-sized penis.^6^ Exposure to media and internet materials has contributed to unrealistic expectations in recent years.^7^^,^^8^ This has led to feelings of inadequacy and discomfort among men not at peace with their bodies.^9^ Various studies have shown that nearly half of men are dissatisfied with the size of their penises and are willing to have larger ones, despite meeting normal physical and anatomic parameters.^10^^,^^11^ Aesthetic procedures for male genitalia have become an integral and essential part of aesthetic surgery worldwide, with a global penile enlargement surgery rate of 8.0%.^12^^,^^13^

Widespread negative attitudes toward body and appearance among men have been identified, and negative body attitudes are associated with negative sexual experiences.^14^ Performance anxiety related to feelings of inadequacy is the most common sexual problem experienced by men. Premature ejaculation (PE) stands out as one of the most prevalent sexual dysfunctions in men, affecting all age groups, with a prevalence of 20% to 30%.^15-17^ The prevalence of PE in our country, Turkey, varies between 20% and 25%.^18^^,^^19^ PE affects not only the individual but also the partner, leading to psychological stress and a loss of self-confidence, negatively affecting the quality of life.^17^ Although there are studies on the prevalence of sexual dysfunctions in men in Turkey, these studies report different results, but it is also seen that these problems are common.^20^ In Turkish culture, the penis is a tool for measuring masculinity and masculinity.^21^ Penis size is considered a symbol of masculinity, and having a large penis has been associated with increased self-confidence.^22^ Many Turkish men attach great importance to their penis size. Despite numerous studies on genital self-image in women, limited information exists in the literature regarding men’s attitudes toward their sexual organs and how they affect PE. This study aims to identify factors influencing men’s genital self-image and the relationship between genital self-image and PE.

## Methods

This descriptive and correlational study was conducted at the urology clinic of a state hospital, which serves a potential population of approximately 100 000 to 120 000, with a total population of 78 402. The hospital comprises 11 departments, an emergency service, 112 emergency health services, and 10 dialysis machines. The sample size calculation was determined as 155 individuals for a medium-sized effect, 80% power, and a type I error of 5%.^23-25^ The effect size was calculated from the study results of Hustad et al.^26^ G*Power was used in the sample calculation. A total of 188 men who applied to the hospital's urology clinic constituted the study sample. Inclusion criteria were age between 18 and 60 years, willingness to participate in the study, and no mental disability. Exclusion criteria were speech and hearing impairments.

### Data instruments

Data were collected by a sociodemographic characteristics questionnaire prepared by the researchers based on relevant literature.^14^^,^^27^ The instruments included the Male Genital Self-image Scale (MGSIS) and the Premature Ejaculation Diagnostic Tool (PEDT).

#### Sociodemographic characteristics form

The sociodemographic characteristics form was created by the researchers through a literature review. It covered participants’ demographics such as age, education status, marital status, family type, employment status, financial status, and income, as well as lifestyle factors such as smoking and alcohol consumption. Additionally, it included questions related to sexual functions, such as the age of first sexual activity, circumcision status, and frequency of sexual intercourse.^14^^,^^27^

#### Male Genital Self-image Scale

The MGSIS, consisting of 7 items to assess feelings and beliefs about male sexual organs, is based on a 4-point Likert scale. Higher total scores indicate a more positive genital self-image. The scale is valid and reliable for measuring men aged 18 to 60 years.^28^ Items are scored from 1 (strongly disagree) to 4 (strongly agree). Total high scores indicate more positive genital self-image. The items of the scale determine satisfaction with the genital area and the level of comfort during the examination of health professionals. Cronbach’s alpha reliability coefficient was calculated as α = 0.92. The scale has no cutoff value or inverse items.

#### Premature Ejaculation Diagnostic Tool

The PEDT is a brief diagnostic measure to assess PE. The short self-administered PEDT, developed by Symonds et al,^29^ was employed. Turkish validity and reliability were established by Serefoglu et al, with a Cronbach’s alpha coefficient of 0.77. The questionnaire comprised 5 items: ejaculation control (items 1 and 3), frequency of PE (item 2), minimum sexual stimulation (item 3), anxiety (item 4), and interpersonal difficulty (item 5). Response options are given on a Likert scale with scores between 0 and 4. Higher values indicate more PE symptoms. The validated 5-item Turkish version of the PEDT is a reliable questionnaire to screen PE among Turkish patients. The scale has no cutoff value or inverse items.^30^

### Data collection

Data were collected by 1 of the researchers in an empty room at the urology clinic of the state hospital, utilizing face-to-face interview techniques that lasted an average of 15 to 20 minutes. After study information was provided, written consent was obtained, ensuring participant privacy. Participants were informed of their right to withdraw from the study at any time, and questions were answered in a comfortable environment.

### Ethical aspect of the research

Approval was obtained from the Health Sciences Scientific Research Ethics Board of Necmettin Erbakan University (2023/456). Ethical approval was also obtained from the relevant authorities of the state hospital. Permissions were secured from the authors who developed the scales.

### Statistical evaluation

Data analysis was performed with SPSS version 25 (IBM). For continuous variables, statistics such as maximum and minimum values, mean, and SD were calculated. For categorical variables, numbers and percentages were computed. Parametric and nonparametric tests were applied according to the normal distribution properties of the data.

## Results

The average age of the men participating in the study was 39.5 ± 9.79 years (mean ± SD), and 91.5% were married. The average age of the participants at their first sexual intercourse was 20.43 ± 4.01 years, and 38.3% stated that they experienced PE. The total mean score of the participants on the MGSIS was 21.10 ± 5.59, and that on the PEDT was 6.96 ± 3.73. It was determined that genital self-image level was significantly lower for participants who had PE problems, were not at peace with their bodies, and were dissatisfied with their sexual lives and genital size and function (*P* < .05; Table 1).

**Table 1:** Sociodemographic characteristics, MGSIS, and PEDT.^a^

			**MGSIS**		**PEDT**	
	**No.**	**%**	**Mean ± SD**	***t* (*P* value)**	**Mean ± SD**	***t* (*P* value)**
Overall sample						
Age, y (range, 18-60)			39.5 ± 9.79			
Age of first sexual intercourse, y			20.43 ± 4.01			
Marriage time, y			15.16 ± 9.59			
MGSIS			21.10 ± 5.59			
PEDT			6.96 ± 3.73			
Relationship status				–1.899 (.059)		0.685 (.494)
Married	172	91.5	20.86 ± 5.55		7.02 ± 3.74	
Single	16	8.5	23.62 ± 5.63		6.33 ± 3.65	
Working status				0.982 (.327)		0.078 (.938)
Yes	166	88.3	21.24 ± 5.49		6.97 ± 3.72	
No	22	11.7	20.00 ± 6.34		6.90 ± 3.91	
Financial situation anxiety				–0.130 (.897)		1.687 (.093)
Yes	89	47.3	21.04 ± 5.21		7.45 ± 3.75	
No	99	52.7	21.15 ± 5.93		6.53 ± 3.68	
Smoking				0.315 (.753)		–1.093 (.276)
Yes	98	52.1	21.22 ± 5.67		6.68 ± 3.67	
No	90	47.9	20.96 ± 5.53		7.27 ± 3.80	
Alcohol				0.336 (.737)		0.356 (.722)
Yes	20	10.6	21.50 ± 3.72		7.25 ± 3.22	
No	168	89.4	21.05 ± 5.78		6.93 ± 3.80	
Premature ejaculation				**–3.752 (<.001)**		**9.508 (<.001)**
Yes	72	38.3	19.22 ± 5.21		9.66 ± 3.14	
No	116	61.7	22.26 ± 5.52		5.27 ± 3.02	
Watching porn				0.826 (.410)		0.251 (.802)
Yes	40	21.3	21.75 ± 4.82		7.10 ± 4.13	
No	148	78.7	20.92 ± 5.78		6.93 ± 3.63	
Comparing himself to others				0.133 (.894)		1.139 (.256)
Yes	25	13.3	21.24 ± 4.58		7.76 ± 3.62	
No	163	86.7	21.07 ± 5.74		6.84 ± 3.75	
Masturbation status				–0.797 (.427)		1.609 (.109)
Yes	83	44.1	20.73 ± 5.31		7.46 ± 3.82	
No	105	55.9	21.39 ± 5.82		6.58 ± 3.63	
Be at peace with his body				**1.964 (.051)**		**–2.635 (.009)**
Yes	141	75.0	21.56 ± 5.73		6.55 ± 3.68	
No	47	25.0	19.72 ± 4.96		8.19 ± 3.65	
Satisfaction with sexual life				**3.023 (.003)**		**–4.651 (<.001)**
Yes	79	42.0	22.55 ± 6.32		5.55 ± 3.78	
No	107	56.9	20.00 ± 4.73		8.00 ± 3.37	
Anxiety of not being able to satisfy the partner				–1.738 (.084)		1.197 (.233)
Yes	25	13.3	19.32 ± 5.68		7.80 ± 3.90	
No	162	86.2	21.40 ± 5.55		6.83 ± 3.70	
Genital size satisfaction				**2.031 (.044)**		**–2.459 (.015)**
Yes	132	70.2	21.63 ± 5.87		6.53 ± 3.70	
No	56	29.8	19.83 ± 4.67		7.98 ± 3.65	
Satisfaction with sexual function organ				**3.417 (.001)**		**–4.300 (<.001)**
Yes	142	75.5	21.74 ± 5.88		6.32 ± 3.58	
No	46	24.5	19.10 ± 4.02		8.93 ± 3.53	

Abbreviations: MGSIS, Male Genital Self-image Scale; PEDT, Premature Ejaculation Diagnostic Tool.

a Bold indicates *P* < .05.

A correlation was found between genital self-image and PE (*r* = –0.238, *P* < .05). In general, a correlation between 0 and 0.25 indicates a very weak negative relationship (Table 2).

**Table 2:** Correlation Between MGSIS and PEDT.

	**MGSIS and PEDT**
Pearson correlation	–0.238
*P* value (2-tailed)	.001
No.	188

Abbreviations: MGSIS, Male Genital Self-image Scale; PEDT, Premature Ejaculation Diagnostic Tool.

Factors affecting men’s PE and genital self-image scale scores were evaluated by multiple linear regression analysis. A significant relationship was found between (1) watching porn movies, thinking about PE, and satisfaction with sexual life and (2) PE and genital self-image (Table 3).

**Table 3:** MGSIS and PEDT: Evaluation of Variables Affecting Scale Scores per Regression Analysis.^a^

	**β**	***t***	***P* value**	**95% CI**	
**Model 1: PEDT** ^**b**^					
Watching porn movies	1.057	1.929	**˂.001**	–0.024	2.137
Thinking about ejaculating prematurely	–4.181	–8.763	**˂.001**	–1637	4953
Sexual life satisfaction	0.705	3.037	.003	0.247	1.164
*R* = 0.609, *R*^2^ = 0.371, Durbin-Watson = 2006 (*P* < .001)					
**Model 2: MGSIS** ^**c**^					
Watching porn movies	–1.828	–1.883	.028	–3.308	–0.116
Thinking about ejaculating prematurely	2.786	3.293	.002	1.024	4.577
Sexual life satisfaction	–0.993	–2.411	.027	–1.849	–0.095
*R* = 0.336, *R*^2^ = 0.113, Durbin-Watson = 1670 (*P* < .001)					

Abbreviations: MGSIS, Male Genital Self-image Scale; PEDT, Premature Ejaculation Diagnostic Tool.

a Bold indicates *P* < .05.

b Model 1: The effect of descriptive variables on PEDT score values.

c Model 2: The effect of descriptive variables on MGSIS score values

## Discussion

In this study, the relationship between PE and genital self-image was determined, and a correlation, albeit weak, was found. A significant difference occurred between those dissatisfied with genital size/function and PE. Generally, low genital self-image is more prevalent in the general population than expected, with a short penis known to signify a weaker genital self-image in men.^26^ For some men, penis size and appearance can affect their self-confidence and be crucial for sexual pleasure.^31^ Concerns about body image during sexual activity are present in men and women, with genitals being the most focused body part for men.^32^ While research indicates that a majority of men report dissatisfaction with their penis sizes, genital image satisfaction encompasses more than just penis size.^33^ Specifically, genital self-image should also consider comfort levels when genitals are seen by health care providers and sexual partners, as well as perceptions of genital appearance and function.^28^

Our study determined that men with poor genital self-image perception experience PE, dissatisfaction with genital size and function, lack of body acceptance, and dissatisfaction with their sexual experiences. Research suggests that negative body image perceptions and sexual anxiety can increase the risk of various sexual dysfunction issues.^2^^,^^33^ For instance, researchers have linked more anxious or avoidant body image with less favorable sexual functioning.^33^^,^^34^ The World Health Organization describes PE as “the inability to delay ejaculation sufficient to enjoy lovemaking, which is manifested by either an occurrence of ejaculation before or very soon after the beginning of intercourse or ejaculation occurring in the absence of sufficient erection to make intercourse possible.”^35^ In our study, the prevalence of PE among participants is up to 38%, but this is only their perception. PE cases require an interprofessional team approach, including physicians, specialists, mental health professionals, specialty-trained nurses, and pharmacists, collaborating across disciplines to achieve optimal patient results.^36^ Considering the presence of various factors influencing PE, the importance of a multidisciplinary approach becomes evident.

According to our study findings, factors such as watching porn movies, thinking about ejaculating prematurely, and sexual life satisfaction were identified as factors affecting PE and genital self-image. Particularly, the influence of media can lead to unrealistic expectations. The rapidly growing porn industry can influence individuals’ subconscious minds, posing a risk especially for those prone to self-criticism and feelings of inadequacy. Inability to fully accept oneself can lead to dissatisfaction and, consequently, sexual problems. It is worth noting that this forms a kind of vicious cycle: unrealistic expectations will lead individuals to blame and punish themselves, which in turn can lead to PE. Sexual dysfunctions are complex and reflect intertwined conditions such as diseases, biopsychosocial factors, treatment side effects, psychological distress, and body image perceptions.^37^ Therefore, it would not be incorrect to emphasize that body image is an important underlying factor affecting sexual function. This study highlights the importance of considering sexual functionality issues, particularly genital self-image, and stresses the significance of taking into account the psychological contribution of genital self-image to PE.

## Conclusion

As a result, it is essential to consider men’s genital self-image as a factor influencing PE. Particularly when individuals experiencing sexual problems are being evaluated, taking into account genital self-image is an important aspect to consider.

## Data Availability

The data sets are available from the corresponding author upon reasonable request.
